# An Overview of Outpatient and Inpatient Detoxification

**Published:** 1998

**Authors:** Motoi Hayashida

**Affiliations:** Motoi Hayashida, M.D., Sc.D., is an Honorable Professor of Psychiatry at Keio University, Tokyo; adjunct professor of psychiatry at University of Pennsylvania, Philadelphia; and medical director of Yokohama Maioka Psychiatric Hospital, Tokyo, Japan

Alcohol detoxification can be defined as a period of medical treatment, usually including counseling, during which a person is helped to overcome physical and psychological dependence on alcohol (Chang and Kosten 1997). The immediate objectives of alcohol detoxification are to help the patient achieve a substance-free state, relieve the immediate symptoms of withdrawal, and treat any comorbid medical or psychiatric conditions. These objectives help prepare the patient for entry into long-term treatment or rehabilitation, the ultimate goal of detoxification (Swift 1997). The objectives of long-term treatment or rehabilitation include the long-term maintenance of the alcohol-free state and the incorporation of psychological, family, and social interventions to help ensure its persistence (Swift 1997).

Alcohol detoxification can be completed safely and effectively in both inpatient and outpatient treatment settings. This article describes the advantages and disadvantages of inpatient and outpatient detoxification programs and considers the influence that the detoxification setting may have on long-term treatment outcomes.

## Outpatient and Inpatient Detoxification

Patients receiving outpatient detoxification treatment usually are expected to travel to a hospital or other treatment facility daily (excluding weekends) for treatment sessions. The sessions may be scheduled for daytime or evening hours, depending on the program. The initial assessment, including intake history, physical examination, ordering of laboratory studies, and the initiation of detoxification treatment, usually takes 1 to 2 hours on the first day of outpatient detoxification. Subsequent sessions may range from 15 to 30 minutes. If the detoxification program is combined with a day hospital program, sessions can last several hours per day. The duration of treatment may range from 3 to 14 days. In one study, the average duration of treatment for outpatients was 6.5 days, significantly shorter than the average duration for inpatient detoxification (i.e., 9 days) (Hayashida et al. 1989). Patients receiving inpatient care are admitted to a hospital or other facility, where they reside for the duration of treatment, which may range from 5 to 14 days.

The process of detoxification in either setting initially involves the assessment and treatment of acute withdrawal symptoms, which may range from mild (e.g., tremor and insomnia) to severe (e.g., autonomic hyperactivity, seizures, and delirium) (Swift 1997). Medications often are provided to help reduce a patient’s withdrawal symptoms. Benzodiazepines (e.g., diazepam and chlordiazepoxide) are the most commonly used drugs for this purpose, and their efficacy is well established (Swift 1997). Benzodiazepines not only reduce alcohol withdrawal symptoms but also prevent alcohol withdrawal seizures, which occur in an estimated 1 to 4 percent of withdrawal patients (Schuckit 1995).

Anticonvulsant medications are necessary in addition to benzodiazepines for patients with a history of seizures unrelated to alcohol withdrawal (Sellers and Naranjo 1986). Additional components of alcohol detoxification may include education and counseling to help the patient prepare for long-term treatment, attendance at Alcoholics Anonymous meetings, recreational and social activities, and medical or surgical consultations.

## Advantages of Outpatient Detoxification

For patients with mild-to-moderate alcohol withdrawal syndrome, characterized by symptoms such as hand tremor, perspiration, heart palpitation, restlessness, loss of appetite, nausea, and vomiting, outpatient detoxification is as safe and effective as inpatient detoxification but is much less expensive and less time consuming (Hayashida et al. 1989). In addition, patients who enroll in long-term outpatient rehabilitation treatment following detoxification in an outpatient setting may benefit by attending the same treatment facility for both phases of treatment. Most outpatients experience greater social support than inpatients, with the exception of outpatients in especially adverse family circumstances or job situations. Outpatients can continue to function relatively normally and maintain employment as well as family and social relationships. Compared with inpatients, those patients in outpatient treatment retain greater freedom, continue to work and maintain day-to-day activities with fewer disruptions, and incur fewer treatment costs.

## Disadvantages of Outpatient Detoxification

Among the drawbacks associated with outpatient detoxification is the increased risk of relapse resulting from the patient’s easy access to alcoholic beverages. In addition, outpatients can more easily choose not to keep their detoxification appointments and, consequently, fail to complete detoxification. In one study of 164 patients randomly assigned to either inpatient or out-patient detoxification, significantly more inpatients than outpatients completed detoxification (Hayashida et al. 1989). The higher completion rate among inpatients should not be interpreted as an indicator of long-term sobriety, however. Inpatients who successfully completed detoxification might have either dropped out of treatment or returned to drinking had they been treated in the outpatient setting. Thus, although inpatients may be more likely to complete detoxification, they may be arbitrarily postponing the chance to resume drinking after discharge.

Outpatient detoxification is not appropriate for all patients. Most alcohol treatment programs find that fewer than 10 percent of patients with alcohol withdrawal symptoms will need admission to an inpatient unit (Abbott et al. 1995). Outpatient detoxification is not safe for alcoholics at risk for potentially life-threatening complications of withdrawal, such as delirium tremens, or those with associated medical conditions such as pancreatitis, gastrointestinal bleeding, or cirrhosis. In addition, outpatient detoxification is not appropriate for suicidal or homicidal patients, those with severe or medically complicated alcohol withdrawal, patients in adverse or disruptive family or job situations, or patients who would not be able to travel daily to the treatment facility.

## Advantages of Inpatient Detoxification

Patients for whom outpatient detoxification is not appropriate become candidates for inpatient detoxification. In-patient settings offer the advantages of constant medical care and supervision provided by a professional staff and the easy availability of treatment for serious complications. In addition, such settings prevent patient access to alcohol and offer separation from the substance-using environment.

## Disadvantages of Inpatient Detoxification

The primary disadvantage of inpatient detoxification is its relatively higher cost compared with outpatient alternatives. In addition, inpatient care may relieve patients of personal responsibilities and encourage unnecessary dependence on hospital staff.

## Detoxification and Overall Treatment Outcome

A number of factors should be considered in determining the appropriate detoxification setting for a particular patient. An important consideration is how the setting might influence overall treatment outcome. For each case, treatment professionals must consider whether inpatient or outpatient treatment would contribute more positively to an alcoholic’s recovery process. Little research has been conducted in this area, however, and the studies that have been conducted do not suggest that one detoxification mode is preferable to another for achieving long-term treatment outcomes. In fact, no significant differences in overall treatment effectiveness, as measured by comprehensive outcome measures such as the Addiction Severity Index (ASI), have been reported between inpatient and outpatient programs (Hayashida et al. 1989). In one study, about one-half of all patients randomly assigned to either inpatient or outpatient detoxification remained abstinent 6 months later, irrespective of the program to which they were assigned. In addition, there was no significant difference in the percentage of each group that enrolled in long-term treatment following detoxification (Hayashida et al. 1989). However, one-third to one-half of patients who enter detoxification treatment, whether as inpatients or outpatients, return to alcohol abuse within 6 months (Hayashida et al. 1989).

Treatment outcome may have more to do with patient characteristics than with detoxification settings. McLellan and colleagues (1983) found that among alcoholics assigned to six different substance abuse treatment programs, patients with low psychiatric severity (as measured by the ASI) at treatment intake improved on outcome measures (i.e., medical condition, alcohol use, other drug use, employment, legal status, family relations, and psychiatric status) in every treatment program, whereas patients with high psychiatric severity showed virtually no improvement in any program. In another study, Volpicelli (1992) found that patients who had three or four symptoms (e.g., a history of alcohol-related seizures, a history of delirium tremens, current unemployment, and intoxication at the initial visit) had a 30-percent chance of completing outpatient detoxification, whereas the patients with none of those symptoms had a 95-percent chance of completion.

A significant number of alcoholics do not respond to treatment beyond detoxification. They “dry out” and then return to alcohol abuse. Many alcoholics repeat this cycle a few times and eventually enter long-term rehabilitation treatment. Some, however, continue to repeat this cycle as “detoxloopers” and exhibit the so-called “revolving-door” phenomenon.

## Transition From Detoxification to Rehabilitative Treatment

Interventions used in rehabilitative treatment can be introduced during detoxification to help the alcoholic complete the process and make the transition to long-term treatment. It is important that the interventions be introduced as early as possible. Such interventions can include treatment for psychological, physical, family, and other needs as well as cue exposure— the repeated exposure to the sight, smell, and taste of alcohol without attendant intoxication effects, which diminishes the physiological and subjective responses originally associated with the alcohol cues.

## Conclusion

The respective advantages and disadvantages of inpatient and outpatient detoxification may make one setting more appropriate than the other for a particular patient, but the detoxification setting does not appear to influence overall treatment outcome. A number of questions remain unanswered concerning how to determine when a particular setting will be advantageous for a patient. For example, the greater freedom provided patients in outpatient detoxification may have positive as well as negative consequences. More research is needed before treatment professionals will be able to discriminate between those patients for whom such freedom would be beneficial and those for whom such freedom would be detrimental.
